# Accelerated super-resolution imaging with FRET-PAINT

**DOI:** 10.1186/s13041-017-0344-5

**Published:** 2017-12-28

**Authors:** Jongjin Lee, Sangjun Park, Wooyoung Kang, Sungchul Hohng

**Affiliations:** 1 Department of Physics and Astronomy, Seoul National University, Seoul, 08826 Republic of Korea; 2 National Center for Creative Research Initiatives, Seoul National University, Seoul, 08826 Republic of Korea; 3 Institute of Applied Physics, Seoul National University, Seoul, 08826 Republic of Korea

**Keywords:** FRET-PAINT, DNA-PAINT, super-resolution microscopy, SMLM, single-molecule localization microscopy, FRET, fluorescence resonance energy transfer

## Abstract

Super-resolution fluorescence microscopy in the current form is hard to be used to image the neural connectivity of thick tissue samples due to problems such as slow imaging speed, severe photobleaching of fluorescent probes, and high background noise. Recently developed DNA-PAINT solved the photobleaching problem, but its imaging speed is extremely low. We report accelerated super-resolution fluorescence microscopy named FRET-PAINT. Compared to conventional DNA-PAINT, the imaging speed of the microscopy increases up to ~30-fold. As demonstrations, we show that 25-50 second imaging time is long enough to provide super-resolution reconstruction of microtubules and mitochondria of COS-7 cells.

**Electronic supplementary material:**

The online version of this article (10.1186/s13041-017-0344-5) contains supplementary material, which is available to authorized users.

## Introduction

Imaging of neural connectivity is challenging because the subcellular structures critical for neural communication—the axon, presynaptic active zone, synaptic cleft, postsynaptic density, and gap junction—are all in tens of nanometers in scale. Serial section electron microscopy, the sole method that is currently available to image neural connectivity with high resolution, is too laborious and error-prone. It does not provide clear pictures of gap junctions, and cannot distinguish whether a chemical synapse is excitatory or inhibitory. Furthermore, it takes huge amount of time to reconstruct a three-dimensional neural connection map from two-dimensional gray-scale image stacks of electron micrographs.

Development of super-resolution fluorescence microscopy has opened a way to study neural structures without being limited by optical diffraction [1–7]. The achievement, however, was not obtained without sacrifice. Compared to conventional fluorescence microscopy, super-resolution fluorescence microscopy techniques usually suffer from aggravated photobleaching and slowed-down imaging speed. Due to these problems, super-resolution fluorescence microscopy in the current form is hard to be directly used to image thick neural tissue samples. Recently developed DNA-PAINT (Point Accumulation for Imaging in Nanoscale Topography [8]) technique has overcome the photobleaching problem by using transient binding of a fluorescently labeled short DNA strand (imager strand) to a docking DNA strand conjugated to target molecules [9–14]. Since photobleached probes are continuously replaced with a new one, fluorescence imaging can be performed without being limited by photobleaching. Furthermore, DNA-PAINT technique provides more photon numbers than other single-molecule localization techniques, resulting in the best localization precision reported until now [12, 14]. The imaging speed of DNA-PAINT (1-3 frames per hour), however, is extremely slow compared to those of other super-resolution fluorescence microscopy techniques [15]. The slow imaging speed of DNA-PAINT is due to slow binding of the imager strand. Since the binding rate of the imager strand is proportional to the ‘imager’ concentration, an obvious solution to this problem is to use higher imager concentration. In current DNA-PAINT technology, however, the imager concentration cannot be increased more than a few nanomolar because background noise also proportionally increases with the imager concentration.

We here developed DNA-PAINT based on FRET (Fluorescence Resonance Energy Transfer [16]). In this technique that we named FRET-PAINT, the docking strand has two DNA binding sites: one for a donor strand and the other for an acceptor strand. For single-molecule localization, FRET signal of the acceptor is used. Since the acceptor is not directly excited but by FRET, 100 times higher imager (donor and acceptor) concentrations could be used. In this paper, we demonstrated ~30-fold imaging speed increase of FRET-PAINT compared to DNA-PAINT.

## Results

### Characterization of FRET-PAINT

**Fig. 1:**
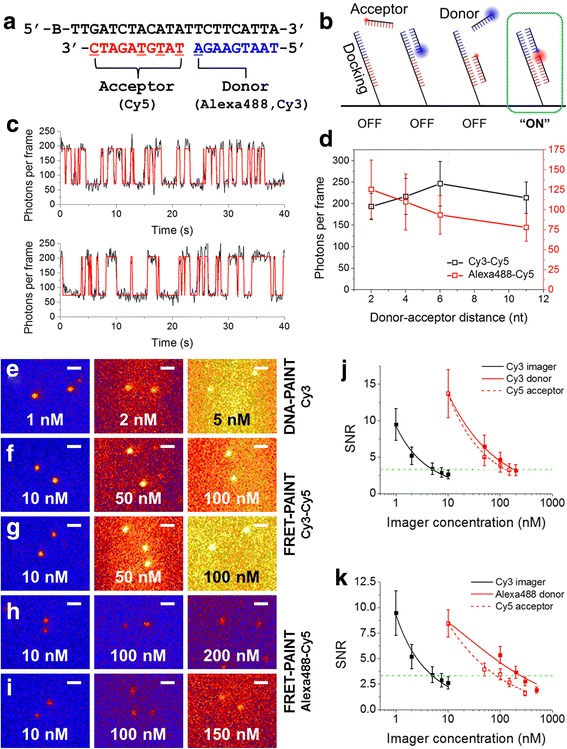
Principle and characterization of FRET-PAINT. **a** Docking (black), donor (blue), and acceptor (red) strands used to characterize FRET-PAINT. The docking strand contains biotin (B) at the 5’-end for surface immobilization. The donor strand is labeled with either Alexa488 or Cy3 at the 3’-end. The acceptor strand is labeled with Cy5 at one of the underlined sites. **b** Scheme of FRET-PAINT. Donor fluorophores are excited but only acceptor fluorophores excited via FRET are detected. **c** Representative Cy5 fluorescence intensity time traces with 1000 nM Donor_P1_Alexa488 and 100 nM Acceptor_P11_Cy5. **d** Normalized FRET efficiency as a function of the donor-acceptor distance for Cy3-Cy5 (black) and Alexa488-Cy5 (red) pairs. (**e**) DNA-PAINT images of surface immobilized docking strand (Docking_P0) at indicated concentrations of Acceptor_P11_Cy3. **f** FRET-PAINT images of Docking_P0 at indicated concentrations of Donor_P1_Cy3 with Acceptor_P6_Cy5 fixed at 10 nM. **g** FRET-PAINT images of Docking_P0 at indicated concentrations of Acceptor_P6_Cy5 with Donor_P1_Cy3 fixed at 10 nM. **h** FRET-PAINT images of Docking_P0 at the indicated concentration of Donor_P1_Alexa488 with Acceptor_P2_Cy5 fixed at 10 nM. **i** FRET-PAINT images of Docking_P0 at the indicated concentration of Acceptor_P2_Cy5 with Donor_P1_Alexa488 fixed at 10 nM. Scale bars: 1 μm. **j** Comparison of SNRs of DNA-PAINT at varying Cy3 imager strand concentration (black) and FRET-PAINT at varying Cy3 donor strand (red solid), and Cy5 acceptor strand (red open) concentration. **k** Comparison of SNRs of DNA-PAINT at varying Cy3 imager strand concentration (black) and FRET-PAINT at varying Alexa488 donor strand (red solid), and Cy5 acceptor strand (red open) concentrations. SNR was defined as the ratio of spot brightness (amplitude of two-dimensional Gaussian fit of spot) to the background fluctuation (FWHM of Gaussian fit of background signal). The data were fitted to an inverse square root of imager concentration. Green dashed lines are added to help find the data points with SNR = 3.3

We first tested the feasibility of FRET-PAINT microscopy using surface-immobilized DNA strands and a total internal reflection fluorescence (TIRF) microscope. In the scheme of FRET-PAINT, three DNA strands—docking, donor, and acceptor strands—are used (Fig. 1a). The docking strand (Docking_P0, Additional file 1) labeled with a biotin at the 5’-end has two docking sites, each of which base-pairs with the donor or acceptor strand. To maintain the photobleaching resistance and high multiplexing capability of DNA-PAINT, both the donor and acceptor strands should be easily replenished with new ones. We used 9 nt donor and 10 nt acceptor strands, which have dissociation rates of 1.2 Hz and 0.02 Hz, respectively (Additional file 1: Figure S1). In this way, photobleached donor and acceptor strands can be continuously replenished by those in the solution. To increase the FRET probability upon donor strand’s binding to the docking strand, we chose a shorter length for the donor strand than for the acceptor strand whereas relatively longer acceptor strand was used. Therefore, the switching speed of FRET-PAINT in our scheme is mainly determined by the dissociation rate of the donor strand. Compared to DNA-PAINT, the length of the docking strand of FRET-PAINT is increased a little bit, but the additional position uncertainty induced by the increased docking strand length is just a few nanometers, which is negligible in most biological applications.

The donor strand was labeled at the 3’-end with Alexa488 (Donor_P1_Alexa488, Additional file 1) whereas the acceptor strand was labeled with Cy5 (Acceptor_P11_Cy5, Additional file 1) at the 3’-end. We immobilized the docking strand on a polymer-coated quartz surface using a streptavidin-biotin interaction and took single-molecule images of Cy5 after injecting the donor (1000 nM) and acceptor (100 nM) strands by exciting Alexa488 using a blue laser. In the scheme, we do not directly excite Cy5 (Fig. 1b), and therefore we could use such high concentrations of donor and acceptor strands without worrying about background noise. As Fig. 1c shows, we obtained clear Cy5 fluorescence intensity time traces at such high donor and acceptor concentrations.

Using the same scheme, we tried to find the optimum labeling position of FRET probes that gives maximum FRET signal for two FRET pairs: Cy3-Cy5 and Alexa488-Cy5. The general requirements for FRET pairs are large spectral overlap between donor emission and acceptor excitation for high FRET efficiency and small spectral overlap between donor emission and acceptor emission for low background noise. The Cy5 was chosen as an acceptor due to its superior photophysical properties such as high photostability and brightness. Alexa488 and Cy3 were selected as a donor because they are photostable and their fluorescence spectra do not significantly overlap with that of Cy5. We prepared donor strands labeled with either Cy3 (Donor_P1_Cy3, Additional file 1) or Alex488 (Donor_P1_Alexa488) at the 3’-end, and acceptor strands labeled with Cy5 at varying positions (Acceptor_P2_Cy5, P4_Cy5, P6_Cy5, and P11_Cy5, Additional file 1). The Cy3-Cy5 FRET pair gave the highest Cy5 signal when the gap between donor and acceptor fluorophores was 6 nt, whereas Alexa488-Cy5 FRET pair gave the highest Cy5 signal when the gap was 2 nt (Fig. 1d). We used these optimized labeling schemes for the remaining part of the paper.

In super-resolution fluorescence imaging, HILO (Highly Inclined and Laminated Optical sheet) [17] microscopy is conventionally used. We compared signal-to-noise ratios (SNRs) of DNA-PAINT and FRET-PAINT at varying DNA concentrations in HILO setup. Figure 1e is DNA-PAINT images of surface immobilized docking strand (Docking_P0) at varying imager strand (Acceptor_P11_Cy3, Additional file 1) concentrations. Single-molecule images started to be overwhelmed by background noise when image concentration was above 5 nM. Figure 1f is FRET-PAINT images of the docking strand at varying donor (Donor_P1_Cy3) concentrations with acceptor (Acceptor_P6_Cy5) concentration fixed at 10 nM. Figure 1g is FRET-PAINT images of the docking strand at varying acceptor (Acceptor_P6_Cy5) concentrations with donor (Donor_P1_Cy3) concentration fixed at 10 nM and. Figure 1h is FRET-PAINT images of the docking strand at varying donor (Donor_P1_Alexa488) concentrations with acceptor (Acceptor_P2_Cy5) concentration fixed at 10 nM. Figure 1i is FRET-PAINT images of the docking strand at varying acceptor (Acceptor_P2_Cy5) concentrations with donor (Donor_P1_Alexa488) concentration fixed at 10 nM. These images clearly show that similar SNR can be obtained at much higher imager concentrations in FRET-PAINT compared to DNA-PAINT. For instance, we used 5 nM imager concentration for DNA-PAINT to obtain the 3.3 SNR (Fig. 1j, k). For the same SNR, we could use 180 nM donor and 120 nM acceptor concentrations for the Cy3-Cy5 pair, and 250 nM donor and 90 nM acceptor concentrations for the Alexa488-Cy5 pair, respectively (Fig. 1j, k). Exact sample numbers for these analyses are described in the Methods section and the error bars represent standard error.

### Superresolution imaging with DNA-PAINT and FRET-PAINT

**Fig. 2:**
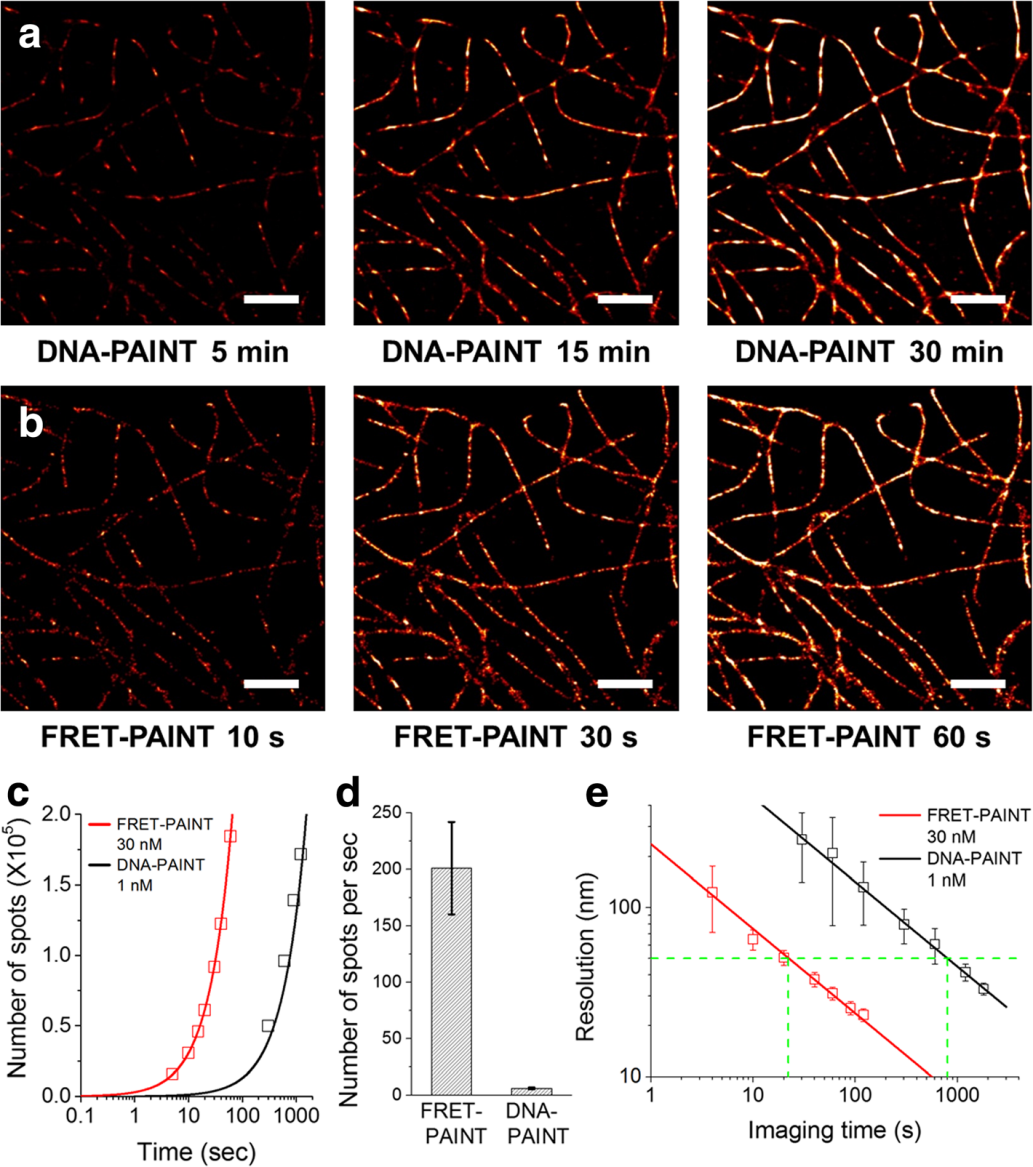
Comparison of the imaging speeds of DNA-PAINT and FRET-PAINT**. a** DNA-PAINT images reconstructed at specified acquisition time. **b** FRET-PAINT images of the same area as in (**a**) reconstructed at specified acquisition time. (**c**) The accumulated number of localized spots as a function of time for DNA-PAINT images of (**a**) (black boxes), and FRET-PAINT images of (**b**) (red boxes). The data are fitted to linear functions (solid lines). The slope of FRET-PAINT is 29-fold larger than that of DNA-PAINT. (**d**) Comparison of the number of localized single-molecule spots per second of FRET-PAINT and DNA-PAINT. Nine different areas were sequentially imaged using FRET-PAINT and DNA-PAINT, and analysed to get the graph. The error bars represent standard deviation. (**e**) Comparison of spatial resolution of DNA-PAINT and FRET-PAINT as a function of imaging time. Seven different imaging areas were analysed to calculate resolution for each image. The error bars represent standard deviation. To obtain 50-nm spatial resolution (horizontal dashed line), 800-s imaging time was required to obtain the same spatial resolution using DNA-PAINT. On the other hand, 22-s imaging time was required for FRET-PAINT when we used 30 nM donor strand, revealing 36-fold increase of imaging speed. Scale bars: 2 um

Next we compared the superresolution imaging speeds of DNA-PAINT and FRET-PAINT. Microtubules of COS-7 cells were immunostained with the anti-tubulin antibody which is labeled with Docking_P1 (Additional file 1). For DNA-PAINT, microtubules were imaged after injecting 1 nM Cy5-labeled imager strand (Acceptor_P2’_Cy5). Single-molecule images were recorded at a frame rate of 10 Hz, which is fast enough to reliably detect binding of donor and acceptor strands (Additional file 1: Figure S1). Figure 2a shows super-resolution images reconstructed at varying acquisition times. Since 18000 frames in total were recorded at a frame rate of 10 Hz for Fig. 2a, the total imaging time was 30 min. For FRET-PAINT, microtubules of the same area were imaged after injecting 30 nM donor (Donor_P1_Alexa488) and 20 nM acceptor (Acceptor_P2_Cy5) strands. Figure 2b shows super-resolution images reconstructed at varying acquisition times. Since 600 frames were recorded at a frame rate of 10 Hz, total imaging time was 60 s. Even by simple eye inspection, it is clear that the speed of FRET-PAINT is much faster than that of DNA-PAINT. To quantitatively compare the imaging speed of DNA-PAINT and FRET-PAINT, we first measured the number of localized spots of Fig. 2a-b as a function of imaging time, and observed 29-fold increase of the imaging speed (Fig. 2c). The same analysis was performed for nine additional imaging areas, and the averaged results are summarized in Fig. 2d, revealing 32-fold increase of the imaging speed on average. As a second way to compare the imaging speeds of DNA-PAINT and FRET-PAINT, we quantified the convolved resolutions computed as ((localization precision)^2^ + (Nyquist resolution)^2^)^1/2^ as previously reported (Fig. 2e) [18]. In our experiments, the localization precisions of DNA-PAINT and FRET-PAINT were 6.9 nm and 11.1 nm, respectively (Additional file 1: Figure S2). Because we used the same excitation laser powers for DNA-PAINT and FRET-PAINT, we think that the little difference in localization precision was caused by the difference in the extinction coefficients of Alexa488 and Cy5. As Fig. 2e shows, the convolved resolution was nicely fitted to a linear function in the log-log plot, indicating that the resolution is mainly determined by Nyquist resolution, which is inversely proportional to the square root of the localization density [19]. By comparing the y-intercept of the two fitting lines, we obtained 36-fold increase of imaging speed.

### Multiplexed imaging with FRET-PAINT

**Fig. 3:**
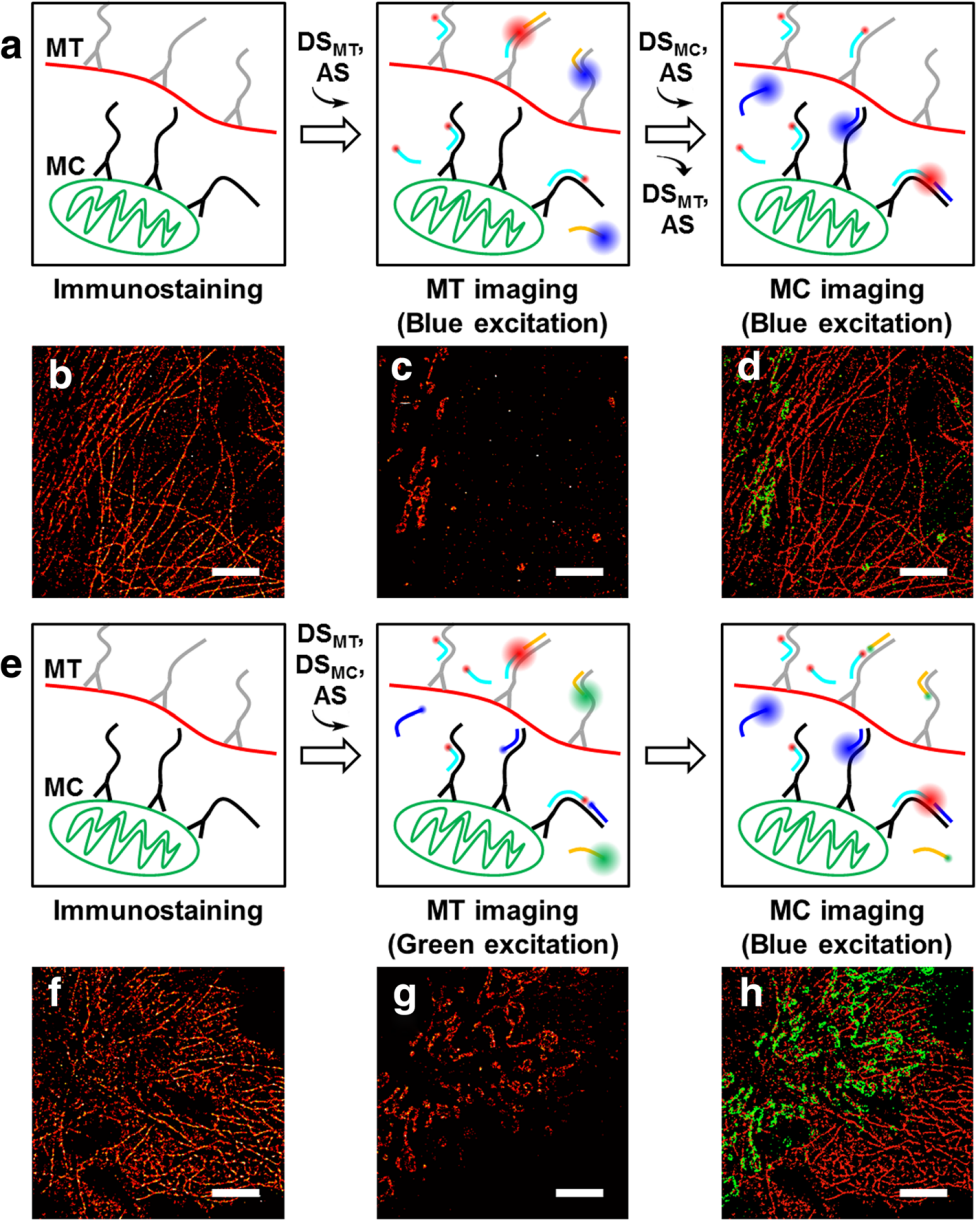
Multiplexing capability of FRET-PAINT. **a** Multiplexed imaging that uses a donor and acceptor strand exchange scheme. FRET-PAINT images of microtubule (**b**), and mitochondria (**c**) obtained using the scheme (**a)**. The both images were obtained at excitation of the same donor (Alexa488) by a blue laser. (**d**) An overlaid image of **b** and **c**. (**e**) Multiplexed imaging without buffer exchange. All donor and acceptor strands are simultaneously introduced into the imaging chamber, but microtubules and mitochondria were imaged sequentially by using different excitation lasers. FRET-PAINT images of microtubule (**f**) and mitochondria (**g**) obtained using the scheme (**e**). Microtubule images were obtained with green laser excitation whereas mitochondria images were obtained with blue laser excitation. (**h**) An overlaid image of **f** and **g**. All FRET-PAINT images were reconstructed from 500 frames recorded at a frame rate of 10 Hz. MT, microtubule; MC, mitochondria; DS, donor strand; AS, acceptor strand. Scale bars: 5 um

Finally, we assessed the multiplexing capability of FRET-PAINT microscopy. Microtubules and mitochondria of COS-7 cells were immunostained using anti-tubulin antibody and anti-Tom20 antibody, respectively. The anti-tubulin antibody and anti-Tom20 antibody were orthogonally conjugated with Docking_P1 and Docking_P2 (Additional file 1), respectively. Two different approaches were used for multiplexed imaging. In the one approach (Fig. 3a), we imaged microtubules first by injecting 20 nM Donor_P1_Alexa488 and 10 nM Acceptor_P2_Cy5 (Fig. 3b) and then imaged mitochondria by injecting 10 nM Donor_P2_Alexa488 (Additional file 1) and 10 nM Acceptor_P2_Cy5 (Fig. 3c). Figure 3d shows an overlaid image of Fig. 3b and Fig. 3c. Spatial organization of microtubules and mitochondria are clearly visualized. In the second approach (Fig. 3e), we injected all DNA probes (10 nM Donor_P1_Cy3 for microtubules, 20 nM Donor_P2_Alexa488 for mitochondria, and 10 nM Acceptor_P2’_Cy5 for both microtubules and mitochondria, Additional file 1) at the same time, and imaged microtubule first with Cy3 excitation (Fig. 3f), and then imaged mitochondria with Alexa488 excitation (Fig. 3g). Figure 3h shows an overlaid image of Fig. 3f and Fig. 3g. Even though the second approach has no advantage in terms of the imaging time, its experimental time was actually decreased because no buffer exchange is required. A disadvantage of the second approach is a cross-talk between microtubule and mitochondria images (Additional file 1: Figure S3). Even though the cross-talk could be partially removed by using intensity filtering, a significant amount of mitochondria were lost during intensity filtering (Additional file 1: Figure S3), demonstrating that the sequential imaging scheme is a better way to do multiplexing imaging.

## Discussion

Despite of several merits of DNA-PAINT, the slow imaging speed of the technique has hindered widespread applications of DNA-PAINT to cellular or tissue imaging. To increase the imaging speed of DNA-PAINT, an obvious solution has been to use higher imager concentrations, but it could not be realized yet due to background noise which proportionally increases with the imager concentration. Here we demonstrated FRET-PAINT can nicely solve the problem and increases the superresolution imaging speed more than ~30-fold. It should be noticed that the advance was achieved without compromising the other advantages of DNA-PAINT: high spatial resolution, photobleaching-resistance, and imaging multiplexing capability. We expect FRET-PAINT will be a useful addition to the advancement of super-resolution fluorescence microscopy.

There are fundamental limits to the imaging speed in single molecule localization based microscopy techniques, which is mainly determined by the switching speed of fluorescence signals. In the scheme of DNA-PAINT and FRET-PAINT, the switching speed can be controlled in principle by changing the binding and dissociation rates of the imager strand. In case of DNA-PAINT, however, the binding rate is hard to be increased more than 2 x 10^-3^ Hz at 2 nM in HILO microscopy due to background noise (Fig. 1, Additional file 1: Figure S1) [20]. We showed that in FRET-PAINT, the biding rate limited by background noise can be increased up to 0.25 Hz at 200 nM imager concentration in HILO microscopy (Fig. 1, Additional file 1: Figure S1). In this paper, however, we could not utilize the full capability of FRET-PAINT for microtubule imaging because single-molecule images started to overlap at 30-nM donor strand concentration (Additional file 1: Figure S4); we used single-emitter localization scheme, and therefore the donor and acceptor strand concentrations used for the FRET-PAINT imaging was determined to avoid the spot overlap. Since the concentration limit dictated by background noise is 10 times higher (Fig. 1f-k, Additional file 1: Figures S5-S6), the imaging speed will be further increased by incorporating shorter donor strand, higher frame rate, and multi-emitter fitting algorithms in the future [21–23].

FRET-PAINT reported in this paper has removed the two main obstacles in the way of using superresolution fulorescence microscopy for three-dimensional reconstruction of thick neural tissue samples: photobleaching of fluorophores and slow imaging speed. However, it is demonstrated only at the cellular level. For FRET-PAINT to be used for neural tissue imaging, huge background noise problem needs to be solved as well. Recently, we developed a real-time confocal microscopy that may provide superresoltion fluorescence images of thick tissue samples using video-rate confocal microscopy for single-molecule imaging [24]. We expect that FRET-PAINT combined with our real-time confocal microscopy will finally enable us to reconstruct three-dimensional structures of thick neural tissue samples with both high speed and high resolution. During the review of our paper in other journals, the exactly same approach to increase the imaging speed of DNA-PAINT was published by Jungmann’s group [25].

## Methods

### Materials

Modified DNA oligonucleotides were purchased from Integrated DNA Technologies. Alexa488 (Alexa Fluor 488 NHS Ester, catalog number: A20000) was purchased from Thermo Fisher Scientific. Cy3 (Cy3 NHS Ester, catalog number: PA13101) and Cy5 (Cy5 NHS Ester, catalog number: PA15101) were purchased from GE Healthcare Life Sciences. COS-7 cells were purchased from Korean Cell Line Bank. Anti-tubulin antibody (catalog number: ab6160) was purchased from Abcam. Anti-Tom20 antibody (sc-11415) was purchased from Santa Cruz Biotechnology, Inc. Donkey anti-rabbit IgG antibody (catalog number: 711-005-152) and donkey anti-rat IgG antibody (catalog number: 712-005-153) were purchased from Jackson ImmunoResearch Laboratories, Inc. Carboxyl latex beads (catalog number: C37281) were purchased from Thermo Fisher Scientific. The docking strands were conjugated to the secondary antibodies using Antibody-Oligonucleotide All-in-One Conjugation Kit (catalog number: A-9202-001) purchased from Solulink. Paraformaldehyde (catalog number: 1.04005.1000) was purchased from Merck. Glutaraldehyde (catalog number: G5882), Triton X-100 (catalog number: T9284), and Bovine Serum Albumin (catalog number: A4919) were purchased from Sigma-Aldrich.

### DNA labeling with fluorophores

Amine-modified DNA oligonucleotides were labeled with fluorophores which have NHS ester chemical group. 5 ul of 1 mM DNA was mixed with 25 ul of 100 mM sodium tetraborate buffer (pH 8.5). And then 5ul of 20 mM fluorophore in DMSO was added. After thorough mixing, the mixture was incubated at 4°C overnight while protected from light. 265 ul of distilled water, 900 ul of ethanol, and 30 ul of 3 M sodium acetate (pH 5.2) were added and mixed thoroughly. The mixture was incubated at -20°C for an hour and then centrifuged for a couple of hours until the DNA pellet is clearly visible. Supernatant was discarded and the pellet was washed with cold ethanol. After ethanol was evaporated completely, the pellet was resuspended in 50 ul of distilled water and the labeling efficiency was measured. If the labeling efficiency is low, the whole labeling process was repeated.

### Cell culture, fixation, and immunostaining

For drift correction of DNA-PAINT imaging, #1.5 glass coverslips were sparsely coated with carboxyl latex beads. The coverslip was coated with bead solution 1:10 diluted in distilled water, heated for 10 minutes on a 100°C hot plate, washed thoroughly with distilled water, and dried with N2 gas. COS-7 cells were grown on bead-coated coverslips for a few days and then fixed for 10 minutes. 2% glutaraldehyde in cytoskeleton buffer was used for microtubule imaging (Fig. 2) and 3% paraformaldehyde and 0.1% glutaraldehyde mixture in PBS buffer was used for microtubule and mitochondria imaging (Fig. 3) [26, 27]. Fixed samples were stored at 4°C in PBS buffer until needed. A flow channel was made by assembling the cell-covered coverslip and a glass slide using double-sided tape and epoxy. In the glass slide, two holes were pre-made for convenient buffer exchange.

Microtubules were immunostained by injecting 1:100 diluted anti-tubulin antibody in blocking solution (5% Bovine Serum Albumin and 0.25% Triton X-100 in PBS buffer) into the channel and incubating at 4°C overnight. After thorough wash-out of free anti-tubulins with PBS buffer, cells were incubated with 100 nM secondary antibody conjugated with docking strand (Docking_P1, Additional file 1) for 1 hour. Mitochondria were immunostained by injecting 1:100 diluted anti-Tom20 antibody in blocking solution into the channel and incubating at 4°C overnight. After thorough wash-out of free anti-Tom20 antibody with PBS buffer, cells were incubated with 100 nM secondary antibody conjugated with docking strand (Docking_P2) for 1 hour.

### Single-molecule imaging

For single-molecule imaging, a prism-type total internal reflection fluorescence (TIRF) microscopy and highly inclined and laminated optical sheet (HILO) microscopy were used. The microscope was built by modifying a commercial inverted microscope (IX71, Olympus), and equipped with a 100X 1.4 NA oil-immersion objective lens (UPlanSApo, Olympus). To obtain data in Fig. 1, docking strands were immobilized on the polymer-coated quartz slide surface by using streptavidin-biotin interaction, and donor and acceptor strands were added into the imaging channel. Alexa488, Cy3, and Cy5 were excited by a blue laser (473 nm, 100 mW, MBL-III-473-100mW, CNI), a green laser (532 nm, 50 mW, Compass 215M-50, Coherent), and a red laser (642 nm, 60 mW, Excelsior-642-60, Spectra-Physics), respectively. Cy3 signal was filtered using a dichroic mirror (640dcxr, Chroma), and Cy5 signal was filtered using a dichroic mirror (740dcxr, Chroma). Single-molecule images were recorded at a frame rate of 10 Hz with electron multiplying charge coupled device (EMCCD) camera (iXon Ultra DU-897U-CS0-#BV, Andor).

### FRET pair characterization

To characterize detected photons per frame in Fig. 1d, 13997 (8096), 11021 (5100), 11208 (3451), and 17051 (3980) single-molecule spots were collected for 2 nt, 4 nt, 6 nt, and 11 nt Cy3-Cy5 (Alexa488-Cy5) FRET pairs, respectively. To characterize SNR in Fig. 1j-k, 795, 2322, and 742 single-molecule spots were collected for Cy3, Cy3-Cy5 pair, and Alexa488-Cy5 pair, respectively.

### Drift correction

For super-resolution imaging with DNA-PAINT, we used a home-made auto-focusing and drift correction system based on image correlation method. Before filming, one in-focus bright field image and two out-of-focus images were taken. These three reference images were used to keep track of x, y, and z axes drift [28]. The drift in z-direction was corrected in real time using a piezo stage (PZ-2000, Applied Scientific Instrumentation) whereas the drift in x-y plane was corrected during image analysis.

## Additional file

Additional file 1: Accelerated super-resolution imaging with FRET-PAINT microscopy. (DOC 6341 kb)
