# Supplementary material for: Accelerated super-resolution imaging with FRET-PAINT
Source: Mol Brain. 2017 Dec 28;10:63. doi: 10.1186/s13041-017-0344-5 (PMC5747120; doi:10.1186/s13041-017-0344-5)
Supplement: Additional file 1: — Accelerated super-resolution imaging with FRET-PAINT microscopy. (DOC 6341 kb) [file 13041_2017_344_MOESM1_ESM.doc]

SUPPLEMENTARY INFORMATION

Accelerated super-resolution imaging with FRET-PAINT microscopy

Jongjin Lee, Sangjun Park, Wooyoung Kang & Sungchul Hohng

| **Supplementary Table** | Sequences and modifications of DNA strands |
| --- | --- |
| **Supplementary Figure 1** | Binding and dissociation rates of donor and acceptor strands |
| **Supplementary Figure 2** | Comparison of localization accuracy of DNA-PAINT and FRET-PAINT |
| **Supplementary Figure 3** | Cross-talk in the multiplexed imaging scheme without buffer exchange |
| **Supplementary Figure 4** | Effect of donor strand concentration on the imaging speed |
| **Supplementary Figure 5** | Effect of donor strand concentration on FRET-PAINT imaging |
| **Supplementary Figure 6** | Effect of acceptor strand concentration on FRET-PAINT imaging |
| **Supplementary References** |  |

| **Name** | **Sequence, modification, labelling position, and description** |
| --- | --- |
| Docking_P0 | - 5’-Biotin-TTGATCTACATATTCTTCATTA-3’ - For surface immobilization |
| Docking_P1 | - 5’-/5AmMC6/TT GATCTACATATTCTTCATTATTTTTTTT-3’ - For microtubule imaging |
| Docking_P2 | - 5’-/5AmMC6/TTGATCTACATATTAACTTTCTTTTTTTTT-3’ - For mitochondria imaging |
| Donor_P1_Amine  Donor_P1_Cy3  Donor_P1_Alexa488 | - 5’-TAATGAAGA/3AmMO/-3’ - 5’-TAATGAAGA-Cy3-3’ - 5’-TAATGAAGA-Alexa488-3’ - Complementary to Docking_P0 and Docking_P1 |
| Donor_P2_Amine  Donor_P2_Cy3  Donor_P2_Alexa488 | - 5’-AGAAAGTTA/3AmMO/-3’ - 5’-AGAAAGTTA-Cy3-3’ - 5’-AGAAAGTTA-Alexa488-3’ - Complementary to Docking_P2 |
| Acceptor_P2_Amine  Acceptor_P2_Cy5 | - 5’-/5AmMO/TATGTAGATC-3’ - 5’-Cy5-TATGTAGATC-3’ - Donor-acceptor distance = 2 nt - 10 nt base-pairing |
| Acceptor_P2’_Amine  Acceptor_P2’_Cy5 | - 5’-/5AmMO/TATGTAGAT-3’ - 5’-Cy5-TATGTAGAT-3’ - Donor-acceptor distance = 2 nt - 9 nt base-pairing |
| Acceptor_P4_Amine  Acceptor_P4_Cy5 | - 5’-TA/iAmMC6T/GTAGATC-3’ - 5’-TA-Cy5-TGTAGATC-3’ - Donor-acceptor distance = 4 nt |
| Acceptor_P6_Amine  Acceptor_P6_Cy5 | - 5’-TATG/iAmMC6T/AGATC-3’ - 5’-TATG-Cy5-TAGATC-3’ - Donor-acceptor distance = 6 nt |
| Acceptor_P11_Amine  Acceptor_P11_Cy3  Acceptor_P11_Cy5 | - 5’-TATGTAGATC/3AmMO/-3’ - 5’-TATGTAGATC-Cy3-3’ - 5’-TATGTAGATC-Cy5-3’ - Donor-acceptor distance = 11 nt |

**Additional file 1: Table S1 Sequences and modifications of DNA strands.**


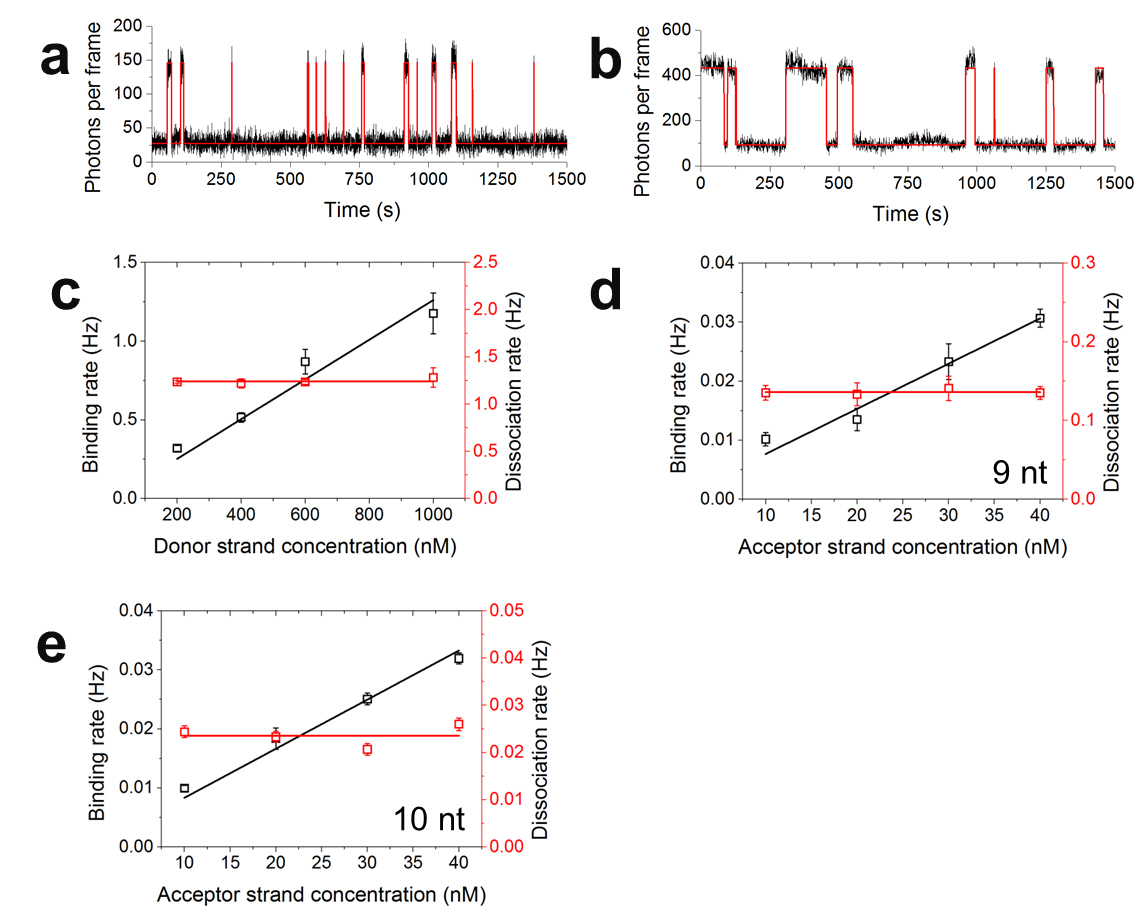


**Additional file 1: Figure S1. Binding and dissociation rates of donor and acceptor strands.**

(**a-b**) Representative binding/dissociation time traces of Acceptor_P2’_Cy5 (**a**) and Acceptor_P2_Cy5 (**b**) at 10 nM concentration. The red lines are added for eye-guide. (**c-e**) Binding (black) and dissociation (red) rates of Donor_P1_Alexa488 (**c**), Acceptor_P2’_Cy5 (**d**), and Acceptor_P2_Cy5 (**e**) as a function of concentration. These binding/dissociation rates are in good agreement with previously reported data [1]. Each data point was obtained from more than 150 binding/dissociation events. The Error bars represent standard error.


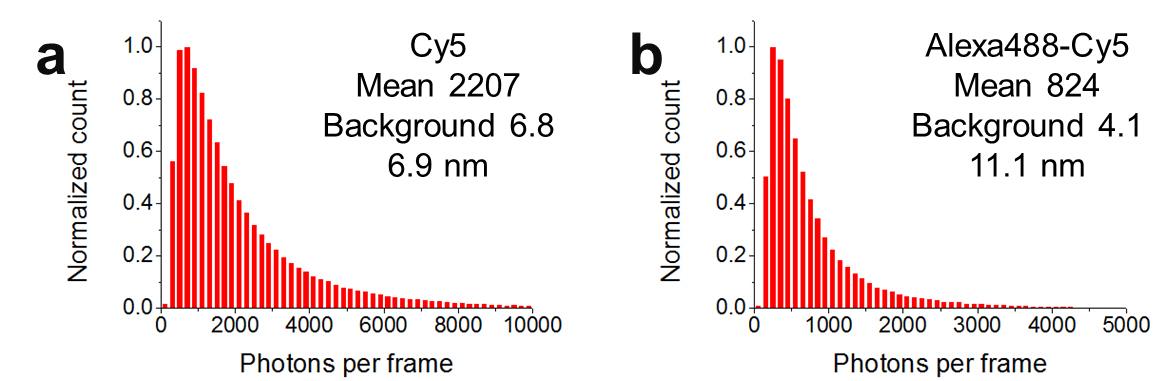


**Additional file 1: Figure S2. Comparison of localization precision of DNA-PAINT and FRET-PAINT.** (**a**) A histogram of the number of photons per frame of single-molecule images that was used to reconstruct Fig. 2a. (**b**) A histogram of the number of photons per frame of single-molecule images that was used to reconstruct Fig. 2b. The localization precision was calculated as previously reported [2]. To calculate the number of photons per frame, 358362 and 182955 single-molecule spots were used for Cy5 and Alexa488-Cy5, respectively.


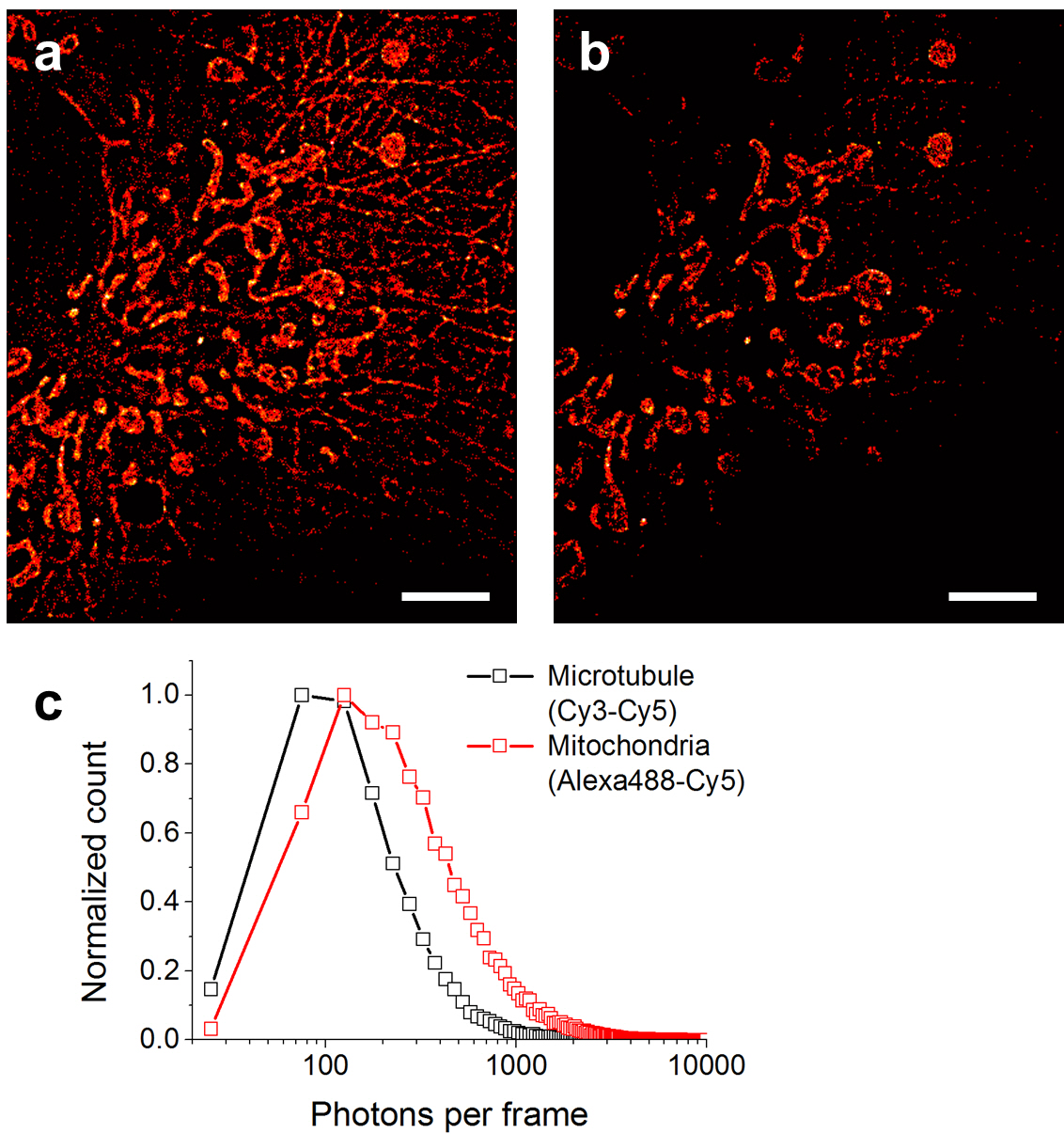


**Additional file 1: Figure S3. Cross-talk in the multiplexed imaging scheme without buffer exchange.**

(**a**) FRET-PAINT image of mitochondria of a fixed COS-7 cell at blue excitation. The image was reconstructed from 500 frames recorded at a frame rate of 10 Hz. The imaging buffer contained not only Donor_P2_Alexa488 for mitochondria but also Donor_P1_Cy3 for microtubule. Even though a 473 nm blue laser was used to excite Alexa488, Cy3 is also excited by some amount, resulting in cross-talk. The cross-talk could be effectively removed by intensity filtering (**b**). Scale bars: 5um. (**c**) Cross-talk between FRET pairs is quantified. At blue excitation, average photon numbers were 680 for Alexa488-Cy5 pair and 290 for Cy3-Cy5 pair. Although the signal of Cy3-Cy5 FRET pair can be removed by intensity filtering, we found that significant amount of Alexa488-Cy5 spots are lost.


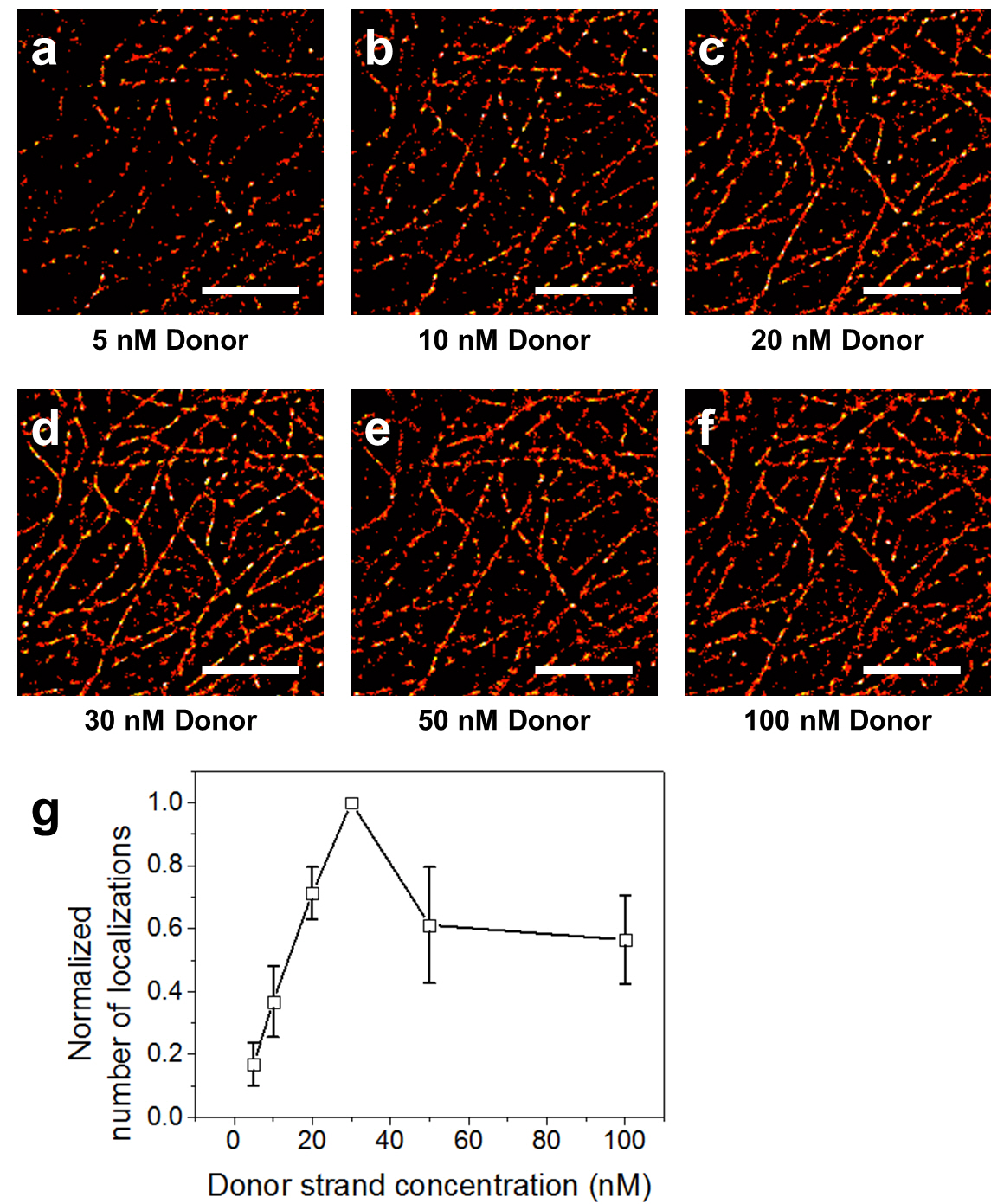


**Additional file 1: Figure S4. Effect of donor strand concentration on the imaging speed.**

(**a-f**) FRET-PAINT microtubule images of a fixed COS-7 cell reconstructed from 200 frames recorded at a frame rate of 10 Hz. The concentration of Donor_P1_Alexa488 was varied as indicated whereas the concentration of Acceptor_P2_Cy5 was fixed at 20nM. Scale bars: 5 um. (**g**) Line/symbol plot of the normalized number of localized spots (open squares) as a function of donor strand concentration. The error bars represent standard deviation of the analysis of six different imaging areas.


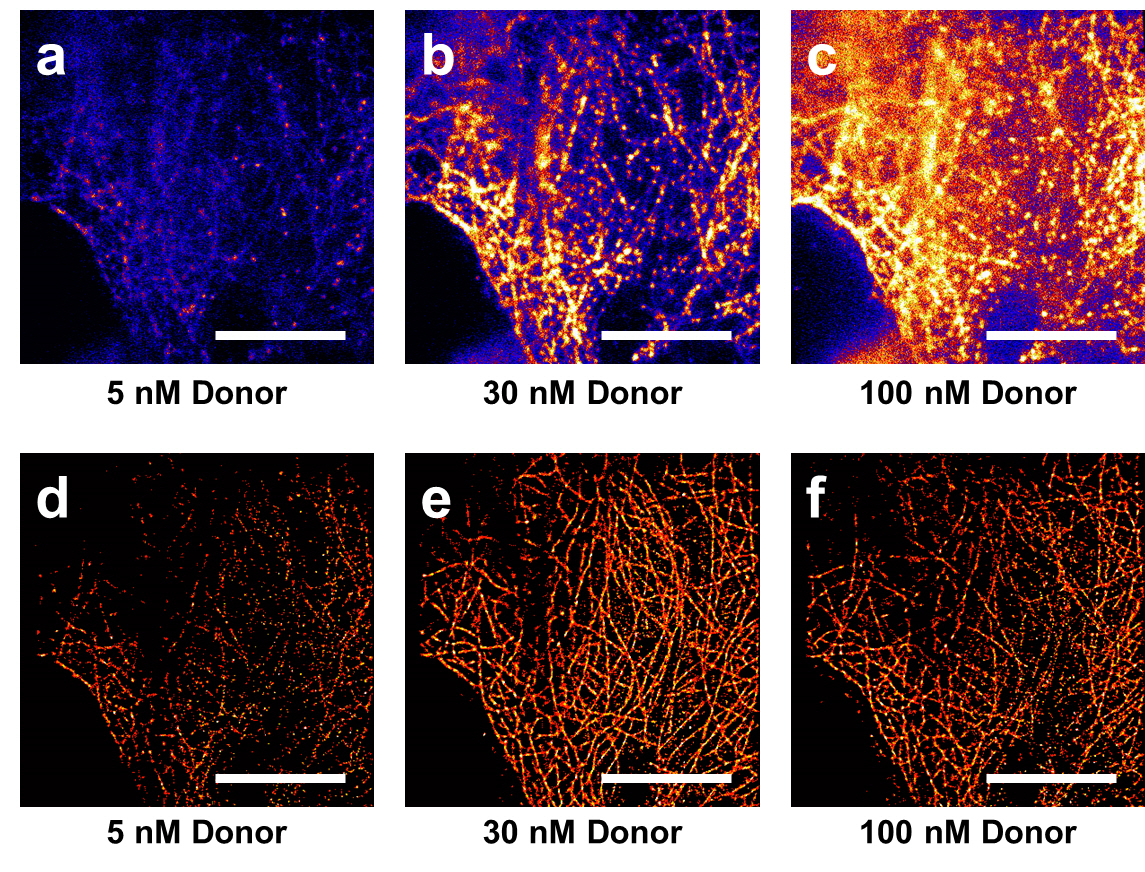


**Additional file 1: Figure S5. Effect of donor strand concentration on FRET-PAINT imaging.** Diffraction-limited raw images (**a-c**) and FRET-PAINT images (**d-f**) of microtubule of a fixed COS-7 cell. FRET-PAINT images were reconstructed from 500 frames recorded at a frame rate of 10 Hz. The concentration of Donor_P1_Alexa488 was varied as indicated whereas the concentration of Acceptor_P2_Cy5 was fixed at 20 nM. By looking at the area outside of the cell of the diffraction-limited raw images, it is evident that background noise is still negligible even at 100 nM donor strand concentration. In our single emitter localization scheme, however, overlap of multiple spots resulted in the decrease of localized spot number above 30 nM donor strand concentration. Scale bars: 10 um.


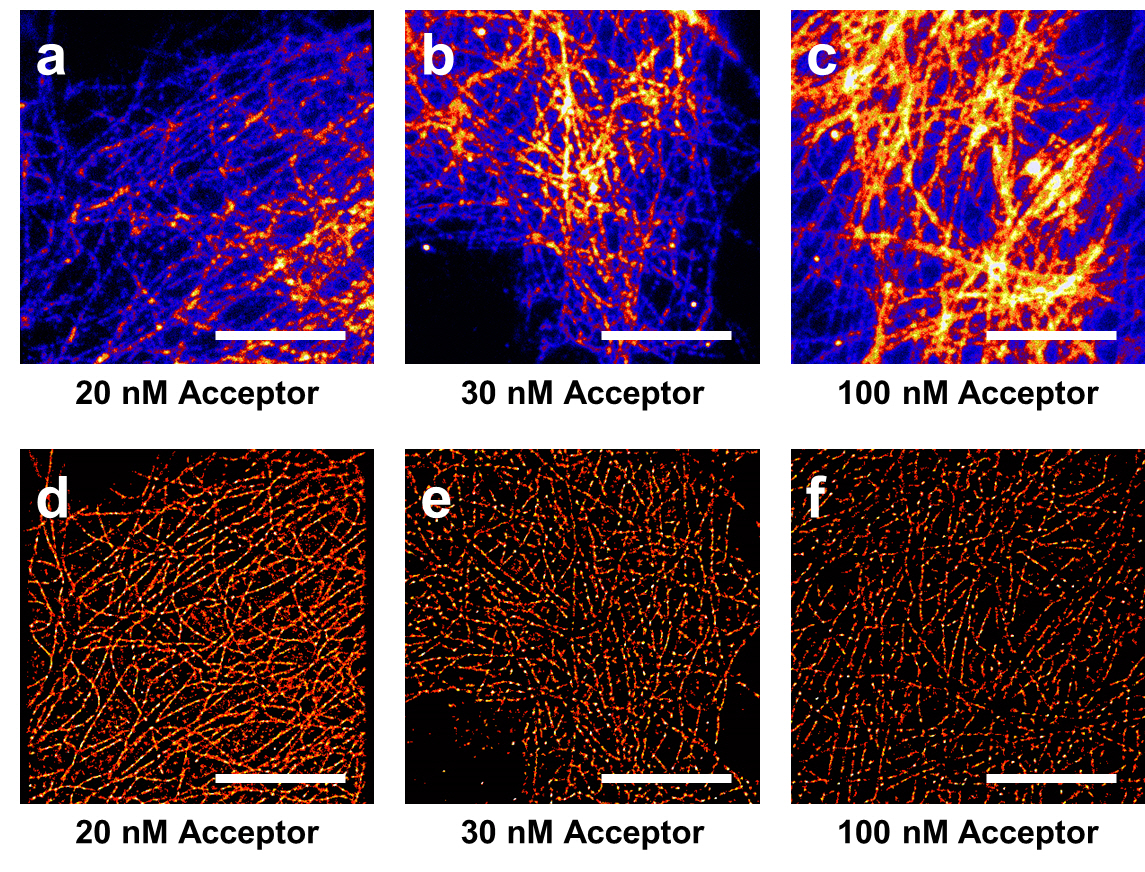


**Additional file 1: Figure S6. Effect of acceptor strand concentration on FRET-PAINT imaging.** Diffraction-limited raw images (**a-c**) and FRET-PAINT images (**d-f**) of microtubule of a fixed COS-7 cell. FRET-PAINT images were reconstructed from 500 frames recorded at a frame rate of 10 Hz. The concentration of Acceptor_P2_Cy5 was varied as indicated whereas the concentration of Donor_P1_Alexa488 was fixed at 30 nM. By looking at the area outside of the cell of the diffraction-limited raw images, it is evident that background noise is still negligible even at 100 nM acceptor strand concentration. In our single emitter localization scheme, however, overlap of multiple spots resulted in the decrease of localized spot number above 20 nM acceptor strand concentration. Scale bars: 10 um.

**Supplementary References**

1. Cisse I, Kim H, Ha T. A rule of seven in Watson-Crick base-pairing of mismatched sequences. Nat. Struct. Mol. Biol. 2012;19:623–7.
2. Thompson RE, Larson DR, Webb WW. Precise nanometer localization analysis for individual fluorescent probes. Biophys. J.2002;82: 2775–83.
